# Droplet Digital PCR Assessment of *MDM2* Amplification in Liposarcoma Diagnosis and Prognosis: A French Single-Center Cohort Study

**DOI:** 10.3390/ijms27156932

**Published:** 2026-08-02

**Authors:** Amira Amri, Aurélie Haffner, Fréderic Fina, Romain Appay, Florence Duffaud, Sébastien Salas, Jean-Camille Mattéi, Alexandre Rochwerger, Christophe Chagnaud, Rémi Fernandez, André Maues de Paula, Pierre-Alexandre Just, Ilyes Hamouda, Shani Diai, Chelsea Anjuly Neda, Patrice Roll, Elise Kaspi, Catherine Gallardo, Anne Barlier, Corinne Bouvier, Diane Frankel, Nicolas Macagno

**Affiliations:** 1 Department of Pathology and Neuropathology, Timone Hospital, Assistance Publique–Hôpitaux de Marseille (AP-HM), Aix-Marseille University, 13005 Marseille, France; aurelie.haffner@ap-hm.fr (A.H.); frederic.fina@ap-hm.fr (F.F.); romain.appay@ap-hm.fr (R.A.); andre.mauesdepaula@ap-hm.fr (A.M.d.P.); pierre-alexandre.just@ap-hm.fr (P.-A.J.); corinne.bouvier2@ap-hm.fr (C.B.); 2 Department of Medical Oncology, La Timone University Hospital, Assistance Publique–Hôpitaux de Marseille (AP-HM), 13005 Marseille, France; fduffaud@ap-hm.fr (F.D.); sebastien.salas@ap-hm.fr (S.S.); 3 Orthopedic and Traumatologic Surgery Department, North Hospital, La Conception Hospital, Timone Hospital, Assistance Publique–Hôpitaux de Marseille (AP-HM), 13005 Marseille, France; jeancamille.mattei@ap-hm.fr (J.-C.M.); richardalexandre.rochwerger@ap-hm.fr (A.R.); 4 Department of Radiology, La Conception University Hospital, Assistance Publique–Hôpitaux de Marseille (AP-HM), Aix-Marseille University, 13005 Marseille, France; christophe.chagnaud@ap-hm.fr (C.C.); remi.fernandez@ap-hm.fr (R.F.); 5 EA 3279, CEReSS, Research Centre on Health Services and Quality of Life, Aix Marseille University, 13005 Marseille, France; ilyes.hamouda@univ-amu.fr (I.H.); shani.diai@univ-amu.fr (S.D.); 6 Cell Biology Department, Timone Hospital, Institut National de la Santé et de la Recherche Médicale (INSERM), Marseille Medical Genetics (MMG), Assistance Publique–Hôpitaux de Marseille (AP-HM), Aix Marseille University, 13005 Marseille, Francepatrice.roll@univ-amu.fr (P.R.); elise.kaspi@univ-amu.fr (E.K.); diane.frankel@ap-hm.fr (D.F.); 7 M2GM, Timone Hospital, Assistance Publique–Hôpitaux de Marseille (AP-HM), 13005 Marseille, France; catherine.gallardo@ap-hm.fr; 8 Laboratory of Molecular Biology GEnOPé, BIOGENOPOLE, Assistance Publique–Hôpitaux de Marseille (AP-HM), Institut National de la Santé et de la Recherche Médicale (INSERM), Marseille Medical Genetics (MMG), MarMaRa Institute, La Timone University Hospital, Aix Marseille University, 13005 Marseille, France

**Keywords:** *MDM2*, ddPCR, liposarcoma

## Abstract

Amplification of *MDM2* is the molecular hallmark of atypical lipomatous tumor/well-differentiated liposarcoma (ALT/WDL) and dedifferentiated liposarcoma (DDL). While fluorescence in situ hybridization (FISH) is widely used for diagnosis, the diagnostic and prognostic value of droplet digital PCR (ddPCR) remains poorly defined. We retrospectively analyzed 341 primary adipocytic tumors, including 85 liposarcomas (22 DDL), using ddPCR to quantify *MDM2* copy number variation (CNV). Diagnostic performance was compared with FISH, and associations with clinicopathological variables and outcomes were evaluated using non-parametric tests, ROC analysis, survival analysis, and Firth’s penalized Cox models. Comparison with FISH confirmed the diagnostic utility of ddPCR for detecting *MDM2* amplification, while quantitative CNV assessment provided additional prognostic information. CNV values were significantly higher in deep-seated and dedifferentiated tumors and were strongly associated with local recurrence. ROC analysis identified a threshold of 16.85 copies predicting both dedifferentiation and recurrence (AUC 0.982 and 0.942, respectively). Patients with CNV ≥ 16.85 copies had significantly shorter recurrence-free and overall survival. In multivariable analysis, high CNV remained an independent predictor of recurrence (HR 36.6, 95% CI 4.0–4914.2). These findings support ddPCR as a robust method for *MDM2* assessment and suggest that quantitative *MDM2* copy number may improve both diagnosis and risk stratification in liposarcoma.

## 1. Introduction

The 2020 WHO Classification of Tumors of Soft Tissue and Bone recognizes several subtypes of lipogenic tumors, which are molecularly distinct and differ in their prognostic implications [1,2,3]. Amplification of *MDM2* represents the defining molecular hallmark of the most common subtype of liposarcoma [4]. *MDM2*-amplified liposarcoma displays a broad spectrum of histopathological patterns depending on the degree of differentiation, each with specific diagnostic challenges and prognostic implications. Well-differentiated liposarcoma (WDL) is primarily characterized by a propensity for local recurrence, whereas dedifferentiated liposarcoma (DL) carries a significant risk of metastasis [5].

While dedifferentiated liposarcoma (DDL) is microscopically indistinguishable from other high-grade sarcomas [6,7,8], the clinico-radiological context combined with strong nuclear *MDM2* immunoreactivity provides critical diagnostic clues, particularly when residual lipogenic areas are present in the histopathological specimen or evident on imaging studies.

Conversely, the most differentiated form, atypical lipomatous tumor/well-differentiated liposarcoma (ALT/WDL), may closely mimic a common lipoma in its lipoma-like subtype due to extensive adipocytic differentiation, whereas the sclerosing and inflammatory subtypes are more readily recognized. In this setting, testing for *MDM2* amplification is particularly useful, as lipoma-like ALT/WDL may fail to demonstrate nuclear *MDM2* overexpression by immunohistochemistry.

While Immunohistochemistry for *MDM2* can serve as a useful screening tool [9,10,11,12,13,14], its specificity is not perfect. The amplification of *MDM2* can be assessed using several techniques, including fluorescence in situ hybridization (FISH), dual-color dual-hapten in situ hybridization (DDISH), Nanostring, quantitative PCR, comparative genomic hybridization arrays (CGH arrays), DNA or RNA sequencing approaches (whole-exome sequencing [WES], whole-transcriptome sequencing [RNA-seq]), and multiplex ligation-dependent probe amplification (MLPA).

Digital droplet PCR (ddPCR) stands out for its high sensitivity and precision in quantifying molecular alterations, including copy number variation (CNV) [15,16,17,18], particularly in small biopsy specimens or circulating DNA. To date, no cohort-based data have been reported on the detection of *MDM2* copy number variation (CNV) using a digital PCR (dPCR) approach.

This study evaluates the performance of digital droplet PCR in detecting *MDM2* amplification in a large cohort of adipocytic tumors, with the aim of reporting its diagnostic and prognostic value.

## 2. Results

### 2.1. Data, Patients and Design

Between 2010 and 2024, 341 patients with primary tumors were included in this retrospective study. The cohort included 183 men (53.7%) and 158 (46.3%) women with a median age at diagnosis of 62.5 years (range 18–89). The diagnostic evaluation by pathologists integrated histological and molecular data and included 256 lipomas and 85 liposarcomas, of which 22 were dedifferentiated. Recurrence-free survival (RFS) and overall survival (OS) data were available for 272 patients, with a median follow-up of 8 months (range: 1 day to 170 months). The overall local recurrence rate was 2.7% (10/341).

Three patients (0.8%) died from disease-related causes after a median of 5 months (range: 2–34), while two additional patients died from unrelated or unknown causes.

### 2.2. MDM2 CNV Results

Given that diagnostic workflows are primarily based on biopsy samples, we first compared CNV_min_ values between biopsies and their corresponding surgical specimens for 39 available pairs to assess concordance. A high level of consistency and a very strong correlation (r = 0.96) were observed in our cohort. Although minor variations were noted in some highly amplified tumors, these did not affect the overall concordance (Supplementary Table S1). Given this high correlation, subsequent analyses were performed using the CNV_min_ values of biopsies.

**Figure 1:**
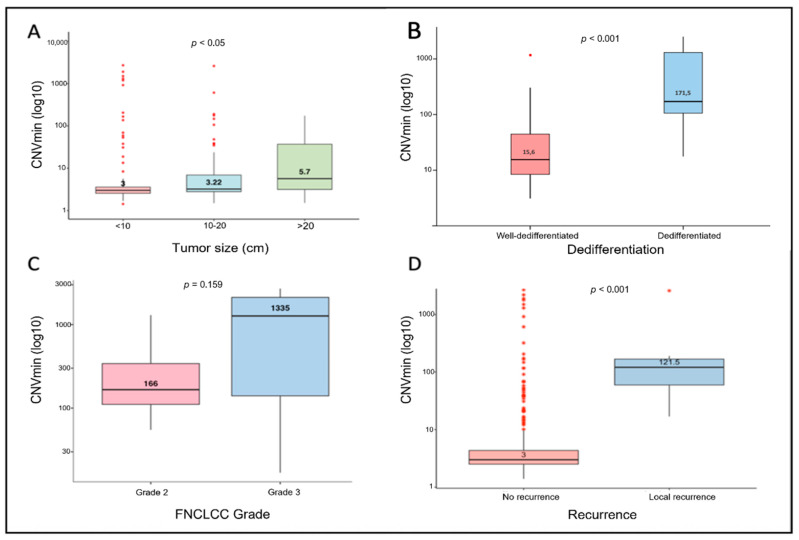
Distribution of CNV_min_ across clinical and pathological variables. (**A**) tumor size (*n* = 84), (**B**) differentiation (*n* = 84), (**C**) FNCLCC grade *(n* = 22), (**D**) CNV_min_ local recurrence (*n* = 10). The numbers displayed within each box correspond to the median CNV_min_ value for each group.

We evaluated the median CNV_min_ values according to several clinically relevant variables. CNV_min_ was higher for tumors larger than 20 cm (median: 5.7 copies) compared to smaller tumors < 10 cm (median: 3.0 copies) and 10–20 cm (median: 3.2 copies, *p* = 0.025, Figure 1A). Similarly, intratruncal tumor sites showed higher CNV_min_ values (median: 13.6 copies) than limbs and extremities (median: 3.0 copies) or other locations (median: 3.1 copies, *p* < 0.001). No significant difference in CNV_min_ values was observed according to patient sex (Table 1, median: 3.1 copies in females vs. 3.1 copies in males, *p* = 0.770).

When analyses were restricted to liposarcomas, the association between tumor size and CNV_min_ remained significant (*p* = 0.042). CNV_min_ also remained higher in intrathoracic and intra-abdominal liposarcomas (median: 59.0 copies) compared to limb tumors (median: 16.1 copies, *p* = 0.005). However, the median observed for other localizations was higher (median: 325.3 copies) compared with the other two groups due to the small sample size. Additionally, dedifferentiated liposarcomas had higher CNV_min_ values (median: 171.5 copies) than well-differentiated tumors (median: 15.6 copies, *p* < 0.001, Figure 1B). Although no differences were found across FNCLCC grades (Fédération Nationale Des Centres de Lutte Contre Le Cancer) (*p* = 0.159, Figure 1C), there was a trend toward higher CNV_min_ values in grade 3 tumors compared to grade 2 tumors.

Finally, patients experiencing local recurrence exhibited markedly higher CNV_min_ values (median: 121.5 copies) on primary biopsy compared to those without recurrence (median: 3.0 copies, *p* < 0.001, Figure 1D).

The detailed clinical and molecular features of the 10 tumors that recurred are summarized in Table 2. Most cases that occurred in deep locations, particularly the retroperitoneum, were dedifferentiated high-grade tumors and exhibited high CNV_min_ levels (>100 copies). Recurrence developed between 7 and 23 months after surgery.

### 2.3. Comparison of ddPCR and FISH for MDM2 Amplification Assessment

**Figure 2:**
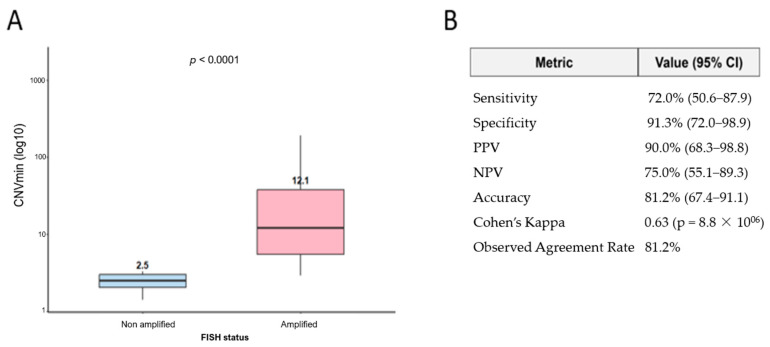
Diagnostic performance of ddPCR compared with FISH. (**A**) Distribution of CNV_min_ values according to FISH status in tumors classified as non-amplified (*n* = 23) or amplified (*n* = 25) by FISH. The numbers displayed within each box correspond to the median CNV_min_ value for each group. (**B**) Diagnostic performance metrics of ddPCR compared with FISH.

To evaluate the diagnostic performance of ddPCR for *MDM2* amplification assessment, CNV_min_ values were compared according to FISH status in the 49 tumors analyzed by both techniques. Tumors classified as amplified by FISH exhibited significantly higher CNV_min_ values than non-amplified tumors (*p* < 0.0001, Mann–Whitney test, Figure 2A).

Using the predefined CNV_min_ threshold (6 copies), ddPCR achieved a sensitivity of 72% and a specificity of 91.3% for the detection of *MDM2* amplification compared with FISH (Figure 2B). Positive and negative predictive values were 90% and 75%, respectively, with an overall accuracy of 81.3%. Substantial agreement between ddPCR and FISH was observed, with a Cohen’s kappa coefficient of 0.63 (*p* = 0.001).

To better understand the discrepancies between the FISH and ddPCR results, we carefully reviewed all cases in which FISH detected an *MDM2* amplification whereas ddPCR yielded a CNV_min_ < 6. Detailed characteristics of these cases are provided in Supplementary Table S2. All seven cases corresponded to core needle biopsy specimens, with a limited amount of tumor tissue providing only limited material for ddPCR analysis. Moreover, FISH-detected *MDM2* amplification involved only a minority of tumor cells (5–50%), suggesting intratumoral heterogeneity. In three cases, the amplification level was low, with a maximum of eight *MDM2* copies per nucleus. Notably, two cases showed borderline ddPCR results (CNV_min_ > 5 and CNV > 6). In addition, surgical resection specimens were subsequently available for five of the seven patients. Reassessment by ddPCR on the resection specimens demonstrated *MDM2* amplification in all five cases (CNV_min_ from 8.5 to 15.7). Histopathological examination confirmed that these tumors were a lipoma-like variant of well-differentiated liposarcomas. Conversely, in one case in which FISH was negative but ddPCR showed a CNV > 6, CGH array analysis confirmed the presence of an *MDM2* amplification.

### 2.4. Predictive Performance of CNV_min_

**Figure 3:**
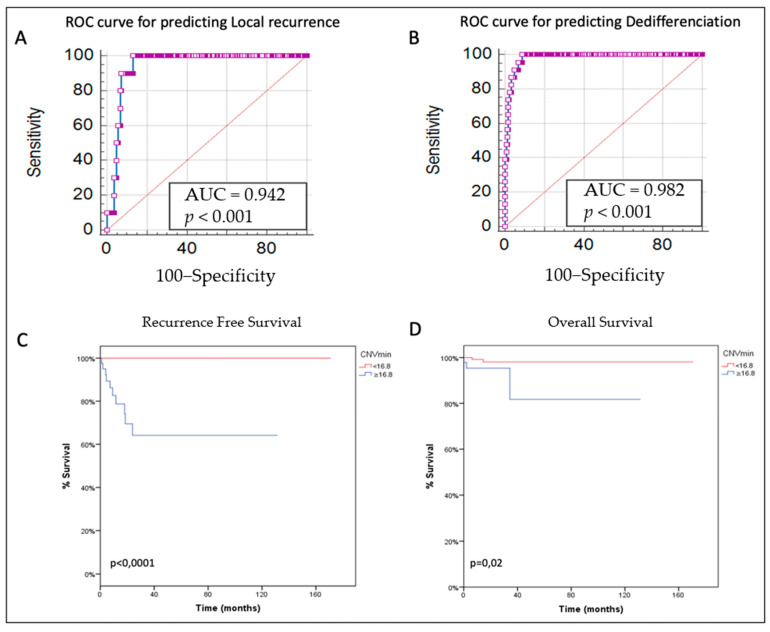
Predictive performance of CNV_min_. ROC curves for CNV_min_ in predicting local recurrence (**A**) and tumor dedifferentiation (**B**). Kaplan–Meier curves for recurrence-free survival (**C**) and overall survival (**D**) stratified by CNV_min_. Survival analysis included 10 local recurrence events and 5 deaths.

To determine the optimal threshold of CNV_min_ for predicting aggressive tumor behavior, ROC curve analysis was performed for both local recurrence and dedifferentiation (Figure 3A,B). The threshold, determined using the Youden index, was 16.85 copies for both, and it effectively discriminated between patients that experienced local recurrence (AUC of 0.942 [95% CI: 0.913–0.968], *p* < 0.001, DeLong test), as well as between well-differentiated and dedifferentiated tumors (AUC 0.982 [95% CI: 0.972–0.996], *p* < 0.001, DeLong test), with 100% sensitivity for both and 86.8% specificity [95% CI: 82.3–90.4] for local recurrence and 90.9% specificity [95% CI: 87.2–93.6%] for dedifferentiation.

Survival analyses were conducted to explore the potential prognostic value of CNV_min_ for recurrence-free survival and overall survival. We divided patients into two groups according to the CNV_min_ threshold of 16.85 copies, previously defined in this same cohort using ROC curve analysis. A shorter recurrence-free survival was found in patients with CNV_min_ ≥ 16.85 copies compared to patients with CNV_min_ < 16.85 copies (*p* < 0.0001, Figure 3C). Similarly, overall survival tended to be lower in the high-amplification group (*p* = 0.02, Figure 3D). High CNV_min_ was associated with shorter recurrence-free survival and shorter overall survival.

To assess whether this effect on recurrence persisted after adjustment for a relevant clinical factor, we performed a multivariable analysis using Firth’s penalized Cox regression, appropriate for the small-event datasets (*n* = 10 recurrences). Intratruncal tumor location was included as a covariate, as it is a recognized adverse factor and showed potential association with recurrence in univariate analysis. In this adjusted model, patients with CNV_min_ ≥ 16.85 copies remained at markedly increased risk of recurrence (HR = 36.6; 95% CI: 4.0–4914.2, *p* = 0.00018, penalized likelihood ratio test), while intra-abdominal or intrathoracic tumor location was also independently associated with recurrence (HR = 7.9; [95% CI 95%: 1.79–76.3], *p* = 0.00425, penalized likelihood ratio test).

## 3. Discussion

In this study, we have shown that ddPCR-based quantification of *MDM2* provided high diagnostic performance and clinically relevant information. To our knowledge, this is the first study to evaluate both the diagnostic and prognostic value of *MDM2* amplification quantified by ddPCR in a large cohort of 341 patients with primary adipocytic tumors.

The most common subtype of liposarcoma is associated with supernumerary ring or giant rod chromosomes resulting from amplified segments on the long arm of chromosome 12 (12q13–15). Among the amplified genes, *MDM2* is the most prominent [19,20], and MDM2 protein inhibits p53, thus promoting sarcoma growth [21,22].

Indeed, in certain situations the molecular diagnosis of ALT, WDL, and DDL relies on confirming the characteristic *MDM2* amplification, which helps to rule out important differentials, notably common lipoma and other sarcoma subtypes. However, *MDM2* amplification can also be observed in other cancers, including both mesenchymal and non-mesenchymal tumors; therefore, its specificity must always be interpreted within the appropriate clinico-radiological and histopathological context. While lipomas do not require follow-up if completely excised, ALT, WDL, and DDL carry a risk of local recurrence, thus necessitating different management and underscoring the importance of an accurate diagnosis. Beyond diagnostic utility, recent data also suggest that amplification levels in the 12q region containing *MDM2*, not only in adipocytic tumors but also in other cancer types, may be associated with prognosis [23].

Historically, FISH was widely recognized as the reference method, and we used it as the gold standard. FISH has, however, several limitations that have been reported, including interpretative subjectivity, technical challenges with degraded or low-cellularity specimens and relatively high implementation costs, particularly in routine diagnostic settings [24]. We observed a substantial agreement between ddPCR and FISH (Cohen’s k = 0.63), with an overall diagnostic accuracy of 81.2%. Using the diagnostic threshold of 6 copies, our results indicate that ddPCR represents a reliable alternative to FISH in appropriate clinical settings for the assessment of *MDM2* amplification, with high specificity and acceptable sensitivity. This threshold is consistent with prior recommendations in molecular diagnostics, where copy number variation between 3 and 5 is interpreted as gains, while values ≥ 6 are considered true amplification [25]. Its technical robustness, ease of standardization, and compatibility with degraded FFPE samples support its potential as a practical and efficient diagnostic tool. Compared with FISH, ddPCR may also offer advantages in terms of workflow standardization, analytical throughput and ease of laboratory implementation.

However, our analysis also highlights some limitations, particularly when the assay is performed on core needle biopsy specimens. Most false-negative ddPCR results occurred in biopsies with limited tumor material and in tumors with heterogeneous *MDM2* amplification, especially the lipoma-like variant of well-differentiated liposarcomas in which *MDM2* amplification is moderate. Therefore, whenever possible, ddPCR should preferentially be performed on surgical resection specimens rather than on biopsy samples. Furthermore, a negative ddPCR result should be interpreted with caution in patients with highly suspicious radiological findings or a strong clinicopathological suspicion of liposarcoma. In such cases, confirmation by FISH remains warranted to avoid false-negative diagnoses. Finally, additional studies focusing on biopsy specimens, particularly from the lipoma-like variant of well-differentiated liposarcomas, are needed to define the minimum amount of tumor material required for optimal ddPCR performance. Future investigations should also evaluate whether the use of frozen tissue instead of FFPE material or pooling multiple biopsy cores could improve the sensitivity of the assay.

Beyond its diagnostic performance, ddPCR-based quantification of *MDM2* also provided clinically relevant prognostic information. Dedifferentiated liposarcomas exhibited markedly higher CNV_min_ values than well-differentiated forms (median: 171.5 and 15.6 copies, respectively, *p* < 0.001—Mann–Whitney). Highly amplified tumors were also predominantly located in intra-abdominal and intrathoracic regions. These findings suggest that the level of *MDM2* amplification may reflect intrinsic tumor aggressiveness and is associated with recurrence-free survival and overall survival. Furthermore, we identified a CNV_min_ threshold of ≥16.85 copies based on ROC analysis of recurrence-free survival, which delineated a subgroup of patients with significantly worse outcomes, including markedly reduced recurrence-free survival (*p* < 0.0001) and decreased overall survival (*p* = 0.02). Notably, this same threshold was also strongly associated with dedifferentiation. Multivariable analysis using Firth’s penalized Cox regression confirmed the independent prognostic value of the CNV_min_ threshold (HR = 36.6; 95% CI: 4.0–4914.2; *p* = 0.00018), even after adjustment for intratruncal tumor location (HR = 7.9). Interestingly, CNV_min_ values were not strongly correlated with tumor size in well-differentiated liposarcomas, suggesting that tumor volume alone does not account for the degree of *MDM2* amplification. In particular, intra-abdominal and intrathoracic forms probably have distinct intrinsic biological characteristics, regardless of their size. This observation is particularly relevant in light of clinical studies evaluating *MDM2*-p53 antagonists, such as milademetan or siremadlin [26,27]. Although these studies did not demonstrate a significant clinical benefit, they underscore the limitations of current therapeutic strategies and the persistent interest in targeting the *MDM2*-p53 axis in liposarcomas.

Of note, only one metastatic patient in our cohort was treated with an *MDM2* inhibitor; therefore, no definitive conclusion can be drawn regarding an eventual correlation between the level of *MDM2* amplification and predictive response to an *MDM2* inhibitor. Interestingly, this patient had a CNV_min_ of 7.3 copies, did not respond well to *MDM2*-inhibition, and died during follow-up. The chemotherapy regimen details are provided in Supplementary Table S3.

However, in this setting, quantification of CNV_min_ by ddPCR could represent a relevant biomarker for risk stratification and potentially for longitudinal monitoring of therapeutic response, especially in advanced cases where conventional imaging and histological assessment may have limitations. Although dedifferentiation can be identified radiologically or histologically, these approaches may be insufficient, especially when based on partial sampling or in morphologically atypical presentations. These results highlight the potential role of *MDM2* amplification quantification as a complementary molecular approach for assessing and monitoring liposarcoma aggressiveness [28] since a biologically plausible dose–response relationship between *MDM2* copy number and clinical outcome can be considered; however, further dedicated studies are required to validate this association.

In addition, the longitudinal dynamics of *MDM2* copy number remain to be investigated. Although our results demonstrate that a high *MDM2* CNV_min_ at diagnosis is associated with an increased risk of recurrence, the present study was not designed to assess temporal changes in *MDM2* copy number within individual patients. Future longitudinal studies based on paired tissue samples and analysis will therefore be required to determine whether temporal variations in *MDM2* copy number reflect tumor evolution, treatment response, or tumor plasticity.

A previous study demonstrated that *MDM2* amplification can be detected in circulating tumor DNA (ctDNA) using shallow whole-genome sequencing [29], highlighting the feasibility of liquid biopsy for monitoring liposarcoma evolution. In this context, ddPCR could represent a promising approach for ctDNA analysis because of its high analytical sensitivity, rapid turnaround time, quantitative accuracy, and ease of implementation in routine molecular diagnostics. Future studies should therefore evaluate the feasibility and performance of ddPCR for *MDM2* copy number quantification in ctDNA and its concordance with tissue-based analysis, particularly for longitudinal monitoring of disease progression and therapeutic response.

Several limitations must be acknowledged. First, the number of local recurrences (*n* = 10) and disease-related deaths (*n* = 3) was relatively small, which may limit the statistical power of our prognostic analysis. This potential bias was mitigated by the use of Firth’s penalized Cox regression model, which is specifically recommended in small-sample settings [30]. Second, the median follow-up duration of 8 months remains relatively short, which may not adequately capture late recurrences. Therefore, the prognostic threshold identified in this study requires external validation in independent cohorts. Finally, although ddPCR is a highly quantitative method, results may vary depending on tumor heterogeneity and the quality of extracted DNA. Despite the use of a standardized protocol, inter-laboratory reproducibility remains to be formally assessed.

Our findings support the integration of *MDM2* quantification by ddPCR in routine practice as a valuable first-line molecular diagnostic tool [31]. On prognostic grounds, patients with CNV_min_ ≥ 16.85 copies could benefit from enhanced follow-up protocols and personalized therapeutic strategies, pending prospective validation. Furthermore, the quantitative nature of ddPCR opens promising perspectives for non-invasive monitoring through liquid biopsy, which may facilitate longitudinal assessment of disease evolution and therapeutic response in dedifferentiated liposarcoma.

## 4. Materials and Methods

### 4.1. Patients

This study included all patients with adipocytic tumors who underwent one or more molecular tests to assess copy number variation in the *MDM2* gene by ddPCR for diagnostic purposes (March 2010 to February 2024) within the Department of Pathological Anatomy and Cytology at La Timone University Hospital. After anonymization and declaration of data usage in compliance with the General Data Protection Regulation (RGPD), the following data were collected: age at first histological diagnosis, sex, tumor size (mm), location, dedifferentiation, recurrence-free survival (RFS), and overall survival (OS). Recurrent disease was detected through imaging techniques and, in a subset of cases, corroborated by histological assessment. RFS was measured in months from the date of the first histological diagnosis to the date of the first local recurrence diagnosis. DSS was calculated similarly, up to the date of death.

### 4.2. DNA Extraction and Quantification

DNA was extracted from sections of FFPE samples. The procedure included deparaffinization using a deparaffinization reagent (DISSOLVR) from the ID-Xtract-MAG-FFPE kit (ID-Solutions, Grabels, France), lysis with proteinase K (PK-FFPE) and a lysis buffer (Lys-FFPE), as well as decross-linking using a specific buffer (D-X-Link). A deparaffinization control was used to verify the absence of contamination. The extraction was performed using the IDEAL^TM^32 automated extraction system (ID-Solutions, Grabels, France). To ensure no contamination during this step, an extraction control (containing extraction reagents without DNA) was included. The extracted DNA was quantified by qPCR using the IDQUANTq-50 kit (ID-Solutions) and the Magnetic Induction Cycler quantitative PCR system (MIC; Bio Molecular Systems). Quantification was performed according to a calibration curve using the positive PCR target control (TPC-IDQUANTq, ID-Solutions, Grabels, France) and the control card provided within the kit. The DNA concentration was adjusted to obtain 8 µL containing 20–200 copies/µL dPCR.

### 4.3. Digital Droplet PCR (ddPCR™)

The ddPCR™ experiments were conducted according to the supplier’s recommendations (Bio-Rad Laboratories, Hercules, CA, USA) using 8 μL of extracted DNA. A PCR reaction mixture was prepared containing probes targeting the *MDM2* gene labeled with a FAM fluorophore (ID copy number assay *MDM2*, Human, dHaCP2500317, Bio-Rad Laboratories) and the reference GAPDH gene labeled with a HEX fluorophore (customized primers and probe by Bio-Rad Laboratories forward, 5′-TGGTAACCTTGTGTCCCTCAATATG-3′ and reverse, 5′-CGCCCCACTTGATTTTGG-3′, a 5′ HEX labeled probe 5′- TCCTGTCCCCATCTCCCCCCC-BHQ-3′), along with ddPCR™ Supermix for Probes (No dUTP, Bio-Rad) and distilled water. The extracted DNA, PCR reaction mixture, and droplet generation oil for probes were loaded into a droplet generator cartridge. The mixtures were partitioned into an emulsion of approximately 15,000 droplets and then transferred onto a PCR plate, which was placed in a thermal cycler (C1000 Touch Thermal Cycler, Bio-Rad). PCR was performed using the following conditions: 95 °C for 10 min; followed by 40 cycles of 94 °C for 30 s and 55 °C for 60 s; and a final extension at 98 °C for 10 min. After amplification, the plate was loaded onto a QX200 droplet reader (Bio-Rad Laboratories). Results were analyzed using QuantaSoft™ Software (version 1.7.4.0917). Every run included a positive control of *MDM2* amplification (a liposarcoma cell line IB111 with a CNV > 40), two cell line negative controls (Calu-6 and H1650 with CNV £ 2) and a negative control with distilled water. The CNV calculated by QuantaSoft™ Software is determined by calculating the ratio CNV = [MDM2]/[GAPDH] × 2 (2 representing the number of copies of reference). GAPDH, a housekeeping gene, was used as an internal reference to normalize for variations in DNA input and to correct for possible cases of polyploidy. For each sample, the minimal CNV value (CNV_min_), corresponding to the lower limit of the 95% confidence interval based on the Poisson distribution, was used to take into account the statistical variability of the CNV calculation. An *MDM2* CNV_min_ threshold ≥6 copies indicates the presence of amplification.

### 4.4. Fluorescence In Situ Hybridization (FISH)

Sections of 3.5 µm thickness were collected on Superfrost slides from FFPE samples. After deparaffinization, rehydration, and pre-treatment steps on the slides using successive baths of xylene, ethanol, deionized water, and a wash buffer solution (Dako), an enzymatic digestion step was performed using pepsin. After dehydration with successive ethanol baths at 70%, 85%, and 100%, a denaturation-hybridization step was carried out by placing the slides on a heating plate (Hybride, ZytoVision, Bremerhaven, Germany) and applying the following probes to the tumor area on the section: *MDM2* (orange spectrum) and centromere of chromosome 12 (CEP12; green spectrum) (Kreatech™, Leica Biosystems, Nussloch, Germany). To allow hybridization, after the denaturation program, which lasted 5 min at 80 °C, the slides were placed in an incubator at 37 °C for 24 h. A washing step followed, and then DAPI II was applied to counterstain the nuclei. Counting was performed on 200 tumor nuclei from the surgical specimen and nearly all the nuclei from the biopsy using the the Metafer 4 image analysis system (MetaSystems, Altlussheim, Germany) with a fluorescence microscope at ×100 magnification. Only nuclei showing both *MDM2* and CEP12 signals were evaluated. Extranuclear signals, poorly visible signals, and overlapping nuclei were not considered. The result was presented as a percentage of analyzed nuclei, specifying the number of green and orange fluorescent spots. The presence of numerous orange spots for two green spots indicated *MDM2* gene amplification. Amplification was considered positive when the number of *MDM2* gene copies exceeded 2 for one CEP12.

### 4.5. Statistics

Descriptive data were provided for the whole sample: quantitative data were expressed as the median and quartiles 1 and 3 and qualitative data were expressed as numbers and percentages. Clinicopathological and prognostic variables were compared according to the CNV_min_ values obtained by ddPCR. Normality was assessed using the Shapiro–Wilk test and Q-Q plots. Parametric tests (Student’s *t*-test for two groups) were applied when assumptions of normality were met; otherwise, non-parametric unpaired tests (Mann–Whitney, Kruskal–Wallis, Spearman correlation coefficient) were used. All statistical tests were two-sided, and the threshold for statistical significance was *p*  =  0.05. The diagnostic performance of CNV_min_, as well as detection of local recurrence or tumor dedifferentiation, was assessed by ROC curve analysis. The area under the curve (AUC) and optimal thresholds were determined using the Youden index. Sensitivity, specificity, positive predictive value (VPP), negative predictive value (VPN) and overall accuracy were calculated with their 95% confidence intervals. Agreement between categorical variables (FISH versus ddPCR) was assessed using Cohen’s kappa coefficient and the observed rate of agreement. Survival curves were calculated according to the Kaplan–Meier method and compared using the log-rank test. Multivariable Cox models were built by including variables that showed a significant association in univariable analysis while limiting the number of covariates according to the number of events. Due to the small number of events (*n* = 10 analyzable local recurrences), a penalized Firth Cox model was used to reduce estimation bias. The results are reported as two-sided *p*-values with 95% confidence intervals (95% CI). Analyses were performed using IBM SPSS Statistics 20 and R (version 4.4.2).

## 5. Conclusions

In conclusion, ddPCR-based quantification of *MDM2* amplification provides a reliable, standardizable, and quantitative approach for the diagnosis and prognostic stratification of primary liposarcomas, supporting its clinical integration. The absolute quantification of *MDM2* amplification may provide additional prognostic value, thus requiring further investigation.

## Figures and Tables

**Table 1:** Association of clinical and histological parameters with CNV_min_.

Variables	*n*	CNV_min_			*p*
		q1	q3	Median	
Sex	341				0.770
Female	158	2.6	6.4	3.1	
Male	183	2.6	4.8	3.1	
Tumor size (cm)	341				0.025
<10	127	2.6	3.6	3.0	
10–20	81	2.8	7.0	3.2	
>20	18	3.0	46.5	5.7	
Missing data	115				
Localization	341				<0.001
Limbs and extremities	205	2.5	6.0	3.0	
Intratruncal	46	3.0	113.8	13.6	
Other	86	2.7	3.3	3,.1	
Missing data	4				
Tumor size (cm) (liposarcoma)	84				0.042
<10	18	27.9	1217.5	103.0	
10–20	22	8.5	116.2	22.1	
>20	9	13.3	79.0	44.3	
Missing data	35				
Localization (liposarcoma)	84				0.005
Limbs and extremities	53	8.2	60	16.1	
Intratruncal	27	30.9	166.0	59.0	
Other	4	24.3	1045.0	325.3	
Differentiation (liposarcoma)	84				<0.001
Well-differentiated	62	8.3	45.3	15.6	
Dedifferentiated	22	96.8	1412.5	171.5	
Grade FNCLCC (liposarcoma)	22				0.159
1/2	11	106.0	610.0	166	
3	10	120.0	2300.0	1335.0	
Missing Data	1				
Local recurrence	341				
Yes	10	58.0	180.2	121.5	
No	288	2.5	4.8	3.0	<0.001
Missing data	43				

**Table 2:** Clinical and molecular features of tumors with local recurrence.

Location	Dedifferentiation	FNCLCC Grade	CNV_min_ (Copies)	Time to Recurrence (Months)
Retroperitoneum	yes	3	16.9	23
Retroperitoneum	yes	2	55	18
Cervico-mediastinal	yes	2	59	4
Intra-abdominal	no	1	61	4
Abdomen	yes	2	106	1
Transverse muscle	yes	3	137	11
Retroperitoneum	yes	1	142	9
Retroperitoneum	yes	2	177	7
Retroperitoneum	yes	2	190	18
Thigh	yes	3	2600	1

## Data Availability

The dataset used and analyzed during the current study is available from the corresponding author upon reasonable request and appropriate ethical approval and data access agreements.

## Supplementary Materials

The following supporting information can be downloaded at: https://www.mdpi.com/article/10.3390/ijms27156932/s1.

## Abbreviations

The following abbreviations are used in this manuscript:

ddPCR	Droplet digital PCR
FISH	Fluorescence in situ hybridization
CNV	Copy number variation
