# Supplementary material for: Droplet Digital PCR Assessment of MDM2 Amplification in Liposarcoma Diagnosis and Prognosis: A French Single-Center Cohort Study
Source: Int J Mol Sci. 2026 Aug 2;27(15):6932. doi: 10.3390/ijms27156932 (PMC13467516; doi:10.3390/ijms27156932)
Supplement: Supplementary file 1 [file ijms-27-06932-s001.zip › Supplementary Table S3.pdf]

**Supplementary Table S3.** Clinical characteristics, treatments, and outcomes of patients with metastatic liposarcoma included in the study. Treatment regimens, including conventional chemotherapy and MDM2 inhibitor therapy, are reported together with MDM2 CNVmin values and clinical outcomes.

| | |
|---|---|
| Head and neck<br>Dedifferentiated liposarcoma Grade II<br>CNVmin 59 | Metastatic Alive with disease<br>Radiotherapy<br>Chemotherapy regimens :<br>Carboplatin |
| Abdomen<br>Dedifferentiated liposarcoma Grade II<br>CNVmin 72 | Metastatic Alive with disease<br>Chemotherapy regimens :<br>Adriamycin |
| Abdomen<br>Dedifferentiated liposarcoma Grade II<br>CNVmin 190 | Metastatic Alive with disease<br>Chemotherapy regimens :<br>Doxorubicin<br>Ifosfamid<br>Taxol<br>Eribulin |
| Dedifferentiated liposarcoma Grade II<br>Abdomen<br>CNVmin 106 | Metastatic Alive with disease<br>Chemotherapy regimens :<br>Endoxan |
| | Metastatic Alive with disease<br>Chemotherapy regimens : Doxorubycine |
| Abdomen<br>Dedifferentiated liposarcoma<br>CNVmin 30 | Metastatic Alive with disease<br>Chemotherapy regimens :<br>Adryamycin<br>Ifosfamide |
| Abdomen<br>Dedifferentiated liposarcoma<br>Grade III<br>CNVmin 177 | Metastatic Alive with disease<br>Chemotherapy regimens :<br>Doxorubicin<br>Eribuline<br>Endoxan |
| Trunk wall<br>Dedifferentiated liposarcoma<br>Grade III<br>CNVmin 137 | Metastatic Alive with disease<br>Eribulin<br>Endoxan |
| Abdomen<br>Dedifferentiated liposarcoma<br>CNVmin 920 | Metastatic Alive with disease<br>Eribulin<br>Endoxan |
| Abdomen<br>Dedifferentiated liposarcoma<br>CNVmin 55 | Metastatic Alive with disease<br>Doxorubycin |
| Abdomen<br>Dedifferentiated liposarcoma grade III<br>CNVmin >1000 | Metastatic Alive with disease<br>Palliative care<br>Eribulin |
| **Abdomen**<br>**Dedifferentiated liposarcoma grade II**<br>**CNVmin 7** | **Dead of Disease**<br>**Metastatic**<br>**Doxorubicin**<br>**Ifosfamid**<br>**Taxol**<br>**Eribulin**<br>**MDM2-inhibitor** |
