# Supplementary material for: Droplet Digital PCR Assessment of MDM2 Amplification in Liposarcoma Diagnosis and Prognosis: A French Single-Center Cohort Study
Source: Int J Mol Sci. 2026 Aug 2;27(15):6932. doi: 10.3390/ijms27156932 (PMC13467516; doi:10.3390/ijms27156932)
Supplement: Supplementary file 1 [file ijms-27-06932-s001.zip › Supplementary Table S1.pdf]

Supplementary Table S1. Clinicopathological characteristics of paired biopsy and surgical resection specimens included in the concordance analysis. Tumor size, anatomical location, and corresponding MDM2 CNVmin values are reported for each paired specimen.

Pair ID	Tumor size (mm)	Anatomical location	Biopsy CNVmin	Surgical specimen CNVmin
122	9	Intratruncal	2.22	3.8
201	45	Intratruncal	2.27	3.73
109	45	Intratruncal	3,0	3,0
392	50	Intratruncal	61,0	234,0
25	60	Limbs and extremities	1.4	1.72
58	60	Limbs and extremities	1.52	3.13
1	60	Limbs and extremities	1.69	2.85
31	60	Limbs and extremities	1.76	3.32
43	70	Limbs and extremities	1.86	2.86
35	85	Limbs and extremities	2.02	1.02
60	90	Limbs and extremities	2.02	2.1
24	100	Limbs and extremities	2.08	1.72
54	100	Limbs and extremities	2.18	2.36
34	105	Limbs and extremities	2.2	3.2
42	110	Limbs and extremities	2.2	1.23
37	110	Limbs and extremities	2.36	2.02
29	120	Limbs and extremities	2.5	2.22
368	130	Limbs and extremities	2.46	4.1
7	135	Limbs and extremities	2.5	3.08
32	140	Limbs and extremities	2.6	2.69
333	150	Limbs and extremities	2.5	2.59
438	150	Limbs and extremities	2.58	2.83
69	160	Limbs and extremities	2.7	2.18
125	170	Limbs and extremities	2.72	5.03
2	170	Limbs and extremities	2.9	5.8
39	170	Limbs and extremities	3.4	5.7
356	170	Limbs and extremities	4.98	7.3
320	180	Limbs and extremities	6.2	6.38
132	200	Limbs and extremities	6.3	18.9
48	200	Limbs and extremities	6.9	3.26
332	200	Limbs and extremities	7.94	11.4
155	220	Limbs and extremities	8.9	13,0
8	260	Limbs and extremities	21.1	16.1
107	280	Head and neck	2.33	3.18
233	290	Head and neck	3.2	2.64
306	350	Trunk	2.52	2.6
234	Unkown	Trunk	3.08	3.56
224	Unkown	Trunk	3.19	3.33
228	Unkown	Trunk	3.6	2.46
