# Supplementary material for: Droplet Digital PCR Assessment of MDM2 Amplification in Liposarcoma Diagnosis and Prognosis: A French Single-Center Cohort Study
Source: Int J Mol Sci. 2026 Aug 2;27(15):6932. doi: 10.3390/ijms27156932 (PMC13467516; doi:10.3390/ijms27156932)
Supplement: Supplementary file 1 [file ijms-27-06932-s001.zip › Supplementary Table S2.pdf]

Supplementary Table S2. Characteristics of discordant cases between fluorescence in situ hybridization (FISH) and droplet digital PCR (ddPCR) for MDM2 amplification assessment. Clinicopathological features, specimen type, FISH findings, ddPCR CNVmin values, and, when available, ddPCR results obtained from the corresponding surgical resection specimens are reported.

**ID**	**sample type**	**location**	**FISH**	**ddPCR (CNV_min)**	**sample type**	**ddPCR (CNV_min)**
44	biopsy	thigh	amplified in 30% of tumoral cells	4,5	surgical resection	10
3	biopsy	deep thigh	amplified in 5% of tumoral cells with 8 to 12 signals	2,86	surgical resection	8.5
39	biopsy	Thigh	amplified in 50% of tumoral cells	3,4	surgical resection	13.7
222	biopsy	retroperitoneum	amplified in all tumoral cells with >20 signals	3,98		
17	biopsy	deep thigh	amplified in 50% of tumoral cells	4,82	surgical resection	15.7
21	biopsy	Thigh	amplified in all tumoral cells	5,4	surgical resection	12
167	biopsy	retroperitoneum	amplified in 20% of tumoral cells with 4 to 8 signals	5,5		
136	biopsy	Thigh	not amplified	6,8		
64	biopsy	shoulder, supra-aponeurotic	not amplified	7,9		
